# Evaluation of Lipid-Based Transfection in Primary Monocytes Within an Ex Vivo Whole-Blood Model

**DOI:** 10.3390/biom15030391

**Published:** 2025-03-08

**Authors:** Robin Moolan-Vadackumchery, Lan Zhang, Frank Stüber

**Affiliations:** 1 Department of Anaesthesiology and Pain Medicine, Inselspital, Bern University Hospital, University of Bern, 3010 Bern, Switzerland; 2 Department for BioMedical Research, University of Bern, 3008 Bern, Switzerland; 3 Graduate School for Cellular and Biomedical Sciences, University of Bern, 3012 Bern, Switzerland

**Keywords:** transfection, ex vivo, RNAi, whole blood, monocytes, Lipofectamine RNAiMAX

## Abstract

Transfection is a fundamental method in biomedical research to study intracellular molecular mechanisms by manipulating target protein expression. Various methods have been developed to deliver nucleic acids into the cells of interest *in vitro*, with chemical transfection by cationic lipids being the most widely used for RNA interference (RNAi). However, translating these *in vitro* results into *in vivo* remains a significant challenge. In this study, we established an *ex vivo* transfection model using cationic lipids in human whole blood. Three different lipid-based reagents were evaluated regarding toxicity, transfection efficiency, and immunogenicity across leukocyte populations using spectral flow cytometry. CD14^+^ monocytes were identified as the primary population to be transfected by cationic lipids in whole blood. To assess immunogenicity, the monocyte-specific activation markers CD80 and human leukocyte antigen DR isotype (HLA-DR) were analyzed upon transfection. Our results demonstrated that Lipofectamine RNAiMAX outperforms the other two reagents, showing low toxicity and high transfection efficiency in combination with a minimal potential for monocyte activation. Functional knockdown experiments using siRNA targeting *CIITA* and the microRNA mir-3972 targeting *HLA-DRA* showed dose-dependent suppression in HLA-DR expression. This study provides the framework for preliminary testing of RNAi in a physiologically relevant *ex vivo* model, enabling assessment of key endpoints such as toxicity, transfection efficiency, and immune activation potential of gene delivery systems.

## 1. Introduction

To investigate complex cellular actions and mechanisms of protein regulation, transient transfection with nucleic acids is a widely used method in immune cell biology. Either plasmid DNA, mRNA, or RNA interference (e.g., by small interfering RNA (siRNA) or microRNA (miRNA)) is used for manipulating target protein expression [1,2]. Chemical, physical, or biological transfection methods are used to introduce nucleic acids into the cells of interest. Among these methods, chemical transfection using cationic lipid formulations is the most popular one for RNA delivery *in vitro* [3]. Cationic lipids consist of three basic components: a cationic head group, a hydrophobic domain, and a linker connecting both parts [4]. The structural composition of the lipid-based carrier molecules determines the physicochemical properties that are crucial for cellular uptake, cytotoxicity, and release of transported nucleic acids into the cytoplasm [5,6]. Cationic lipid transfection involves multiple sequential steps, including particle uptake, membrane fusion, and finally, intracellular cargo release. Upon mixing, the anionic nucleic acids spontaneously complex with the cationic lipid formulation in a highly cooperative manner driven by their opposing charges. The complex formation protects nucleic acids from degradation and prevents unintended interactions with other components in the biological milieu. The resulting lipoplexes, which possess a net positive charge, interact with negatively charged plasma membrane proteins and glycans, facilitating their attachment to the cell surface. This interaction induces the endocytic pathway and results in lipoplex-containing endosomal vesicles. Intracellularly, the lipoplex cargo must exit the endosome before fusion with acidic lysosomes and subsequent degradation [7]. This so-called endosomal escape is considered to be critical for transfection efficiency. Thus, the fusion of lipoplexes with the endosomal membrane is essential for the release of nucleic acids into the cytoplasm. Various studies have demonstrated that the composition and structural characteristics of cationic lipids influence their efficiency in facilitating endosomal escape [8,9]. Chemical transfection with RNA possesses several advantages compared to biological transfection protocols (e.g., viral transduction). First, introduced RNA is processed in the cytoplasm, and therefore, no nuclear entry is required. Second, there is no interference with the genomic DNA of the host cell, which reduces genotoxicity issues [10]. Thirdly, immunogenicity is reduced because no viral components potentially triggering an inflammatory response, are required [11]. Additionally, chemical transfection protocols are usually simple and fast and do not need time-intensive preparation. Compared to physical transfection methods, like electroporation, lipofection is cost-efficient and scalable [12,13]. Due to these benefits, cationic lipid-based vectors also open an interesting path to gene therapy. Successful clinical translation, exemplified in the use of lipid-based nanoparticles in mRNA vaccines for SARS-CoV-2, has been shown [14]. However, transfection efficiency, immunogenicity, cytotoxicity, and stability *in vivo* can vary from *in vitro* findings. Therefore, extensive preliminary *in vivo* experiments are required to identify the best approach to achieve high transfection efficiency and low cytotoxicity [15].

To provide a model closer to physiology than *in vitro* cell lines and to investigate cellular mechanisms on the way to *in vivo* applications, we developed a transient chemical transfection system *ex vivo* using human peripheral blood. The effects of three commercially available cationic lipid formulations for siRNA/miRNA delivery were assessed across leukocyte subsets. Cell viability, transfection efficiency, and immune cell activation were set as critical readouts. These were assessed following transfection with a mock miRNA, while functional knockdown experiments using siRNA and miRNA for targeted protein manipulation demonstrated the model’s effectiveness.

## 2. Materials and Methods

### 2.1. Whole-Blood Transfection Using Three Different Transfection Reagents

Peripheral blood was obtained from healthy individuals using standard butterflies and S-Monovette^®^ Hirudin tubes (Sarstedt, Nümbrecht, Germany) [16]. Within one hour of collection, 162 µL whole blood per well were plated in a flat-bottom 96-well plate (TPP, Trasadingen, Switzerland), and transfection was performed as described below. Written informed consent was obtained from all blood donors.

Transfection of siRNA or miRNA was performed using the commercially available transfection reagents lipofectamine RNAiMAX (Thermofisher Scientific, Waltham, MA, USA), DharmaFECT-4 (Horizon, Lafayette, CO, USA), or HiPerFect (Qiagen, Hilden, Germany) according to the manufacturer’s instructions. Transfection was performed based on manufacturers protocols, and the samples were incubated for 24 h at 37 °C with 5% CO_2._

Lipofectamine RNAiMAX (LipoRM): miRNA/siRNA was diluted in 9 µL OptiMEM (Life Technologies, Carlsbad CA, USA) to reach final concentrations of 5, 10, 15, and 20 nM. LipoRM was diluted in a total volume of 9 µL OptiMEM according to the increasing concentrations of miRNA/siRNA. The respective LipoRM solution was subsequently added to the miRNA/siRNA solution and incubated for 15 min at room temperature (RT). Afterward, 18 µL of the LipoRM-miRNA/siRNA solution were added to 162 µL whole blood (ratio of transfection reagents to whole blood 1:10).

DharmaFECT-4 (Dhar4): miRNA/siRNA solution was prepared for the same final concentrations in 20.25 µL OptiMEM. Transfection reagent was prepared proportional to the increasing concentrations of miRNA/siRNA by diluting Dhar4 in a total volume of 20.25 µL OptiMEM. Both solutions were incubated for 5 min at RT. Afterward, the respective Dhar4 solution was added to the miRNA/siRNA solution and incubated for 20 min at RT. After incubation, 40.5 µL of Dhar4-miRNA/siRNA solution were added to 162 µL whole blood (ratio of transfection reagents to whole blood 1:5).

HiPerFect (HiPe): miRNA/siRNA solution was prepared for the same final concentrations in 11.6 µL OptiMEM. HiPe solution was prepared by diluting transfection reagent in a total volume of 11.6 µL OptiMEM according to the manufacturer’s instruction for the increasing concentrations of miRNA/siRNA. Afterward, the respective HiPe solution was added to the miRNA/siRNA solution, mixed well, and incubated for 10 min at RT. Subsequently, 23.2 µL of the HiPe-miRNA/siRNA solution was added to 162 µL whole blood (ratio of transfection reagents to whole blood 1:8).

After 24 h incubation, culture plates were centrifuged for 3 min at 300× *g*, and the supernatants were replaced by 150 µL Iscove’s modified Dulbecco’s medium (IMDM) containing 2 mM L-Glutamine (Merck, Darmstadt, Germany) [17]. Samples were incubated for a further 48 h before preparation for subsequent experiments.

### 2.2. Two-Step Red Blood Cell Lysis and Sample Preparation for Flow Cytometry

At the end of the experiment, the samples were transferred into a U-bottom 96-well plate (TPP, Trasadingen, Switzerland) and centrifuged for 5 min at 300× *g*. Supernatants were removed, and 180 µL of Cell-Vive CD RBC Lysis Buffer (Biolegend, San Diego, CA, USA) were added to each well and mixed thoroughly. The plates were then incubated for 12 min on a plate shaker at RT. After centrifugation, the supernatants were discarded. For the second lysis step, 60 µL of the RBC lysis buffer were added to the pellets, mixed well, and incubated for 5 min at RT. Lysis was stopped by adding 150 µL cold phosphate-buffered saline (PBS) + 1% fetal calf serum (FCS) (Sigma-Aldrich, St. Louis, MO, USA). The plates were centrifuged for 5 min at 300× *g* at 4 °C. Afterward, the supernatants were removed, and the cells were resuspended in 10% human serum AB type (Sigma-Aldrich, St.Louis MO, USA) for 10 min at 4 °C to avoid FC receptor-involved unspecific antibody binding. Subsequently, the cells were stained with antibodies for 45 min at 4 °C in the dark, washed, and fixed using 0.5% paraformaldehyde (Sigma-Aldrich, St. Louis, MO, USA). Samples were measured using a Cytek Aurora-5L spectral flow cytometer (Cytek Biosciences, Fremont, CA, USA). The analysis and median fluorescence intensity calculation of target proteins were performed using the FlowJo V10 software (BD Biosciences, Franklin Lakes, NJ, USA).

### 2.3. Cell Viability

Samples were prepared as described in the previous section. miRNA *c.el-239b* (CN-002000-01; Horizon, Lafayette, CO, USA) from *Caenorhabditis elegans* without a target in the human transcriptome was used as a mock transfection sequence. For measuring cell viability, a 100 µL 4′,6-diamidin-2-phenylindol (DAPI) solution (Merck, Darmstadt, Germany) at a final concentration of 1 µg/mL was given to the samples for 1 min. After washing, the cells were fixed, and the DAPI-negative live cells and DAPI-positive dead cells were discriminated by spectral flow cytometry.

### 2.4. Transfection Efficiency

Transfection efficiency was measured using a commercially available BLOCK-iT Alexa Fluor Red Fluorescent control kit (Thermofisher Scientific, Waltham, MA, USA). The fluorescent-labeled siRNA was used at final concentrations of 5, 10, 15, and 20 nM in combination with one of the three transfection reagents to assess the transfection efficiency in blood leukocyte subsets. Samples were prepared as described in the section above, and transfected cells were identified by fluorescence intensity of Alexa Fluor 555 using spectral flow cytometry.

In a further step, CD14^+^ monocytes were imaged using a 12-channel Imagestream^®^ MkII imaging flow cytometer (Cytek Biosciences, Fremont CA, USA). Briefly, 400 µL whole blood were transfected with either of the three transfection reagents and BLOCK-iT (final concentration 20 nM), in a 48-well plate (TPP, Trasadingen, Switzerland) and incubated for 24 h. Afterward, the supernatants were discarded and PE-anti-HLA-DR (2 µg/mL) and FITC-anti-CD14 (2.5 µg/mL) antibodies were added to the samples and incubated for 45 min at 37 °C with 5% CO_2_. Subsequently, the red blood cells were lysed, and the remaining cells were fixated by adding 4 mL FACS lysing solution (BD Biosciences, Franklin Lakes NJ, USA) for 5 min. After centrifugation (300× *g*, 5 min), the supernatants were removed, and 400 µL DAPI solution (1 µg/mL) was added to the pellets for 4 min to counterstain the nuclei. Subsequently, cells were washed twice and resuspended in 60 µL cold PBS before images were acquired. Image analysis was performed using IDEAS^®^ software Version 6.3 (Cytek Biosciences, Fremont, CA, USA) to quantify the expression of Alexa Fluor 555 in CD14^+^ HLA-DR^+^ monocytes.

### 2.5. Immune Cell Activation Assay

Monocyte activation was measured by the activation markers CD80 and HLA-DR [18,19]. Whole blood was transfected with *c.el-239b* in combination with one of the three transfection reagents. Samples were prepared as described above and stained with APC-anti-CD80 (2.5 µg/mL) and PE-anti-HLA-DR (1 µg/mL) antibodies. The median fluorescence intensity (MFI) of the respective target proteins was determined using spectral flow cytometry.

### 2.6. Transfection Assay Ex Vivo Using siRNA and miRNA

On-target siRNA *CIITA* (J-011083-07; Horizon, Lafayette CO, USA) and miRNA mimic mir-3972 (C-302158-00; Horizon, Lafayette, CO, USA) were prepared for final concentrations of 5, 10, 15, and 20 nM with LipoRM in OptiMEM medium, and transfection was performed as described above. After the lysis of red blood cells, blood leukocytes were stained with PE-anti HLA-DR (1 µg/mL), FITC-anti-CD16 (2.5 µg/mL) and PerCP-anti-CD14 (2.5 µg/mL). After 45 min incubation at 4 °C in the dark, samples were prepared for spectral flow cytometry. The MFI of HLA-DR expression was analyzed.

### 2.7. Statistical Analysis

GraphPad Prism software version 10.4.1 was used for the statistical analysis and graphing of results. Data are presented as mean ± standard deviation. Statistical analyses were performed using either multiple *t*-tests or one sample *t*-test for normalized data. *p* < 0.05 was considered statistically significant.

### 2.8. Antibodies

PE—anti-CD4(Clone RPA-T4)Pacific Blue—anti-CD4(Clone OKT4)PE-Cy7—anti-CD8(Clone HIT8a)PerCP—anti-CD14(Clone HCD14)FITC—anti-CD15(Clone HI98)FITC—anti-CD16(Clone 3G8)Pacific Blue—anti-CD16(Clone 3G8APC—anti-CD19(Clone HIB19)APC-Cy7—anti-CD56(Clone HCD56)Alexa Fluor 488—anti-HLA-DR(Clone L243)PE—anti-HLA-DR(Clone L243)APC—anti-HLA-DR(Clone L243)APC—anti-CD80(Clone 2D10)

All antibodies were purchased from Biolegend, San Diego, CA, USA

## 3. Results

### 3.1. Viability of Whole-Blood Leukocyte Populations upon Ex Vivo Transfection

**Figure 1:**
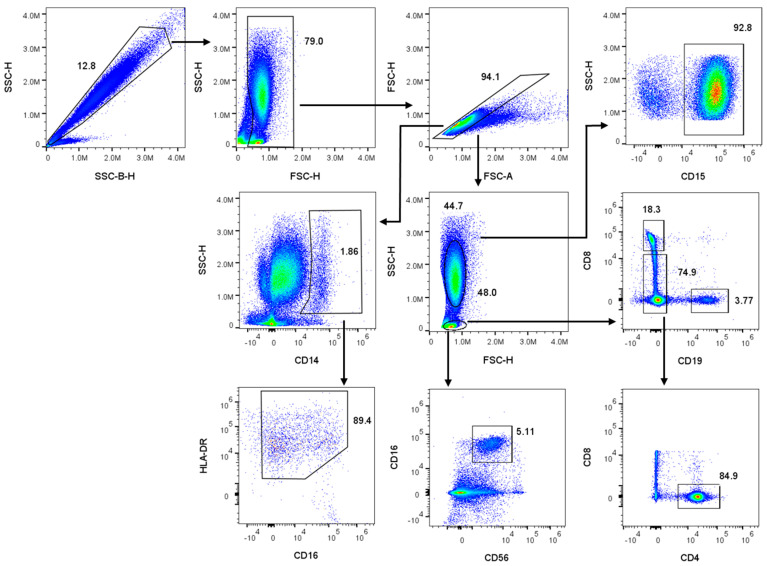
Gating strategy for identification of blood leukocyte populations based on cell-specific CD marker expression.

**Figure 2:**
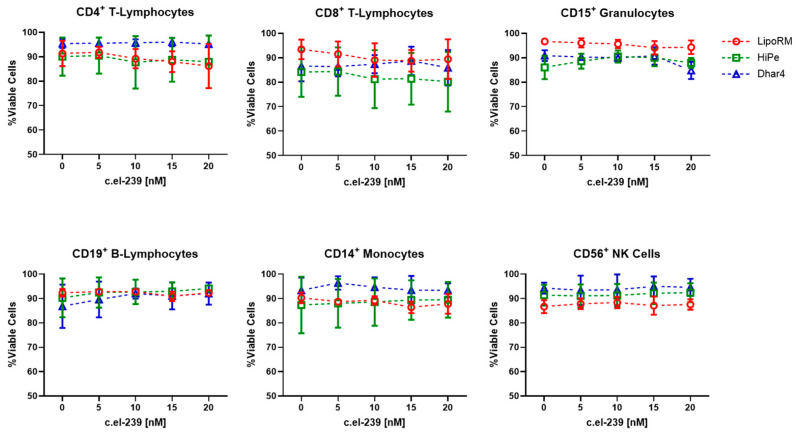
Viability of leukocyte populations after transfection using different transfection reagents. Whole-blood cells were transfected with increasing concentrations of mock miRNA sequence (*c.el-239b*) and one of the three transfection reagents (LipoRM, HiPe, and Dhar4). Percentage of viable cells was determined. No miRNA (0 nM) refers to untransfected samples. Values are presented as mean ± standard deviation (SD); n = 3.

The introduction of exogenous nucleic acids often causes a disturbance in cell homeostasis and eventually might affect viability [20]. For example, cationic lipids can induce cytotoxicity by interaction with cellular membranes and subsequent disruption of membrane integrity [21,22]. Therefore, cell-type dependent optimization of transfection protocols is crucial for the successful insertion of nucleic acids into target cells and subsequent manipulation of protein expression. To investigate the consequences of lipid-based transfection on cell viability in whole blood, we performed flow-cytometric analysis on leukocyte populations including CD4^+^ T-lymphocytes, CD8^+^ T-lymphocytes, CD19^+^ B-lymphocytes, CD15^+^ granulocytes, CD56^+^ natural killer cells, and CD14^+^ monocytes. Whole blood was transfected with three commercially available reagents supposedly made for transfection with miRNA or siRNA. The three reagents were loaded with increasing concentrations (5 nM to 20 nM) of the mock sequence miRNA *c.el-239b*. After 72 h, the erythrocytes were lysed, and the leukocytes were stained with the corresponding CD markers. Immediately before flow cytometric measurement, the cells were treated with DAPI to distinguish between live and dead cells. To identify the populations of interest, a gating strategy, as illustrated in Figure 1, was applied. The remaining erythrocytes were singled out by two different side scatter lasers (SSC vs. SSC-B). Based on their hemoglobin absorption at 405 nm, the red blood cell population shifts due to the reduced SSC signal [23]. After debris exclusion and singlet selection (FSC-H vs. FSC-A), different leukocyte subpopulations were isolated based on their specific CD marker expression. In the final step, DAPI-positive cells, which are considered dead or apoptotic, were identified using cooked cells as a gating control. Among all three transfection reagents, the percentage of viable cells remained consistently above 80%, with minimal variation across all leukocyte populations (Figure 2). Surprisingly, increasing concentrations of reagents and *c.el-239b* did not show a significant decrease in viability, indicating the applicable range for the use of the three carrier reagents.

### 3.2. Transfection Efficiency Across Leukocyte Sub-Populations in Whole Blood

**Figure 3:**
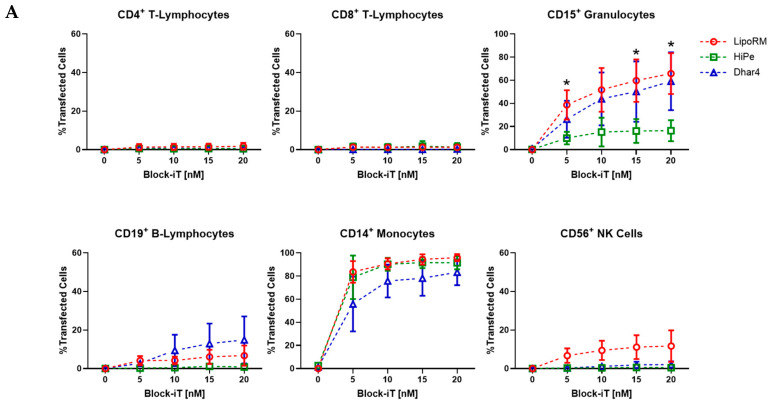

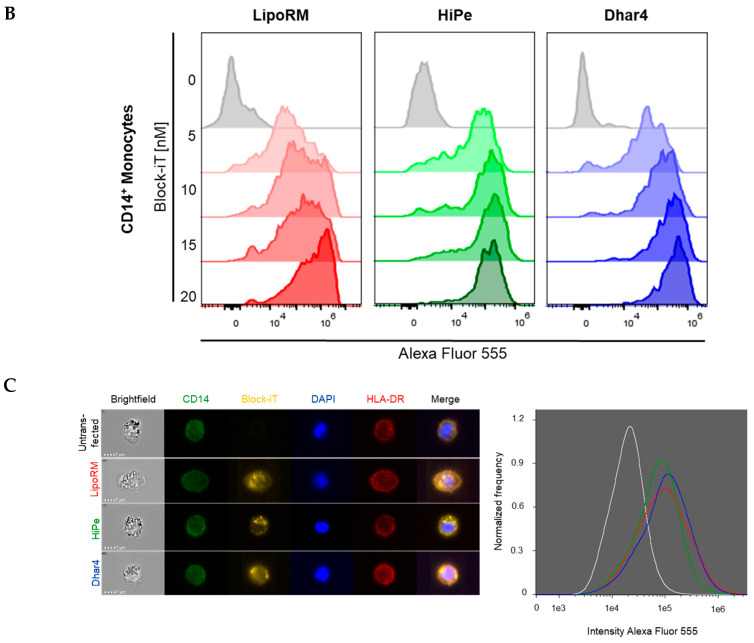
Transfection efficiency in leukocyte populations after using increasing concentrations of Alexa Fluor 555-labeled miRNA (Block-iT) and different transfection reagents. (**A**) Percentage of Alexa Fluor 555 positive cells 72 h post-transfection using LipoRM, HiPe, or Dhar4. Values for no miRNA (0 nM) refer to untransfected cells. Values are presented as mean ± standard deviation (SD); n = 3. Multiple *t*-test; statistical differences in transfection efficiency are depicted with * for LipoRM vs. HiPe, * *p* < 0.05. (**B**) Representative histograms with increasing Alexa Fluor 555 intensity as indicator for positive transfected CD14+ monocytes. (**C**) 60× Representative images and intensity histogram of primary CD14+ monocytes treated with 20 nM Block-iT and different transfection reagents LipoRM (red), HiPe (green), or Dhar4 (blue) acquired by imaging flow cytometry.

Transfection efficiency is crucial for the success of any transfection experiment and depends on various parameters [21]. Transfection efficiency was measured in sub-populations for each transfection reagent. Alexa Fluor 555-labeled miRNA (Block-iT) was used to identify the leukocytes that are responsive to lipid-based transfection by measuring the red fluorescence with spectral flow cytometry. Across all populations, only CD15^+^ granulocytes and CD14^+^ monocytes showed a substantial proportion of Alexa Fluor 555-expressing cells. The percentage of positively transfected CD15^+^ granulocytes increased with the rising concentrations of LipoRM and Dhar4, up to 65.8% ± 14.4% and 59.1% ± 20.4%, respectively, whereas efficiency was significantly lower when HiPe was used (16.4% ± 7.4%) (Figure 3A). In CD14^+^ monocytes, the percentage of transfected cells was already high at 5 nM Block-iT for all three reagents. Using LipoRM and HiPe, transfection efficiencies of 83.6% ± 7.6% and 78.9% ± 15.4% were observed. Dhar4 showed a lower efficiency than the other two reagents, with 55.8% ± 19.3%. With increasing concentrations, the transfection efficiency in CD14^+^ monocytes exceeded 80% for all three reagents (LipoRM: 96% ± 2.5%; HiPe: 91.5% ± 4.6%; and Dhar4: 83.3% ± 9.2%) (Figure 3A,B). In primary lymphocytes, the transfection efficiency consistently remained below 20% (Figure 3A). Particularly, CD4^+^ and CD8^+^ T-lymphocytes exhibited very low Alexa Fluor 555 expression, indicating minimal to no uptake of the lipid complexes. CD19^+^ B-lymphocytes and CD56^+^ NK cells displayed Alexa Fluor 555 expression when treated with Dhar4 and LipoRM, respectively. Treatment with Dhar4 resulted in 14.9% ± 10.0% of CD19^+^ B-lymphocytes being Alexa Fluor 555 positive, while 11.7% ± 6.7% of CD56^+^ NK cells were Alexa Fluor 555 positive following treatment with LipoRM. In addition, the uptake of labeled miRNA and potential morphological changes in monocytes were determined using imaging flow cytometry. The images revealed no significant differences in Alexa Fluor 555 expression among the three transfection reagents (Figure 3C). Taken together, our results demonstrate that nucleic acid delivery in primary leukocytes is moderately efficient in CD15^+^ granulocytes and highly efficient in CD14^+^ monocytes using the selected reagents. In CD15^+^ granulocytes, LipoRM and Dhar4 were favored compared to HiPe, which displayed low transfection efficiency. In the case of CD14^+^ monocytes, the transfection efficiency was high at even low reagent concentrations. Although not statistically significant, slightly lower values were observed when Dhar4 was used. Based on these findings, further experiments focused on primary monocytes.

### 3.3. Immune Activation in Primary Monocytes upon Use of Transfection Reagents

**Figure 4:**
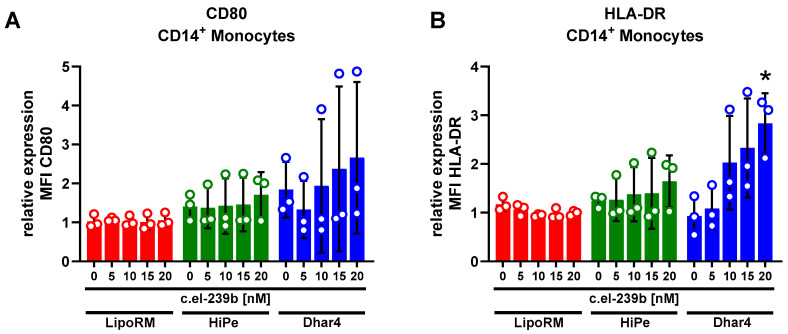
Immune activation of monocytes assessed via HLA-DR and CD80 expression after transfection with LipoRM, HiPe, or Dhar4. (**A**) Median fluorescence intensity (MFI) of CD80 expression after transfection with mock miRNA sequence (*c.el-239b*) normalized to untransfected monocytes. (**B**) MFI of HLA-DR expression after transfection with mock miRNA sequence (*c.el-239b*) normalized to untransfected monocytes. Values for no miRNA (0 nM) refer to cells treated with transfection reagent only. Values are presented as mean ± standard deviation (SD); one sample *t*-test * *p* < 0.05 compared to untransfected cells; n = 3.

One major concern while performing transfection studies is the immunogenicity of lipid-based methods. The introduction of foreign material, such as artificial nucleic acids, can induce pro-inflammatory functions and/or lead to the apoptosis of transfected cells [24]. Therefore, the potentials of the three transfection reagents to trigger an immune response were investigated for primary monocytes. The expressions of monocytic activation markers CD80 and HLA-DR were measured 72 h post-transfection with mock sequence *c.el-239b* and plotted as an x-fold increase compared to untransfected primary monocytes in Figure 4A,B. Treatment with LipoRM resulted in the least impact on CD80 and HLA-DR expression. The levels remained consistently low, even with increasing concentrations of *c.el-239b* up to 20 nM. Transfection with Dhar4 displayed elevated CD80 and HLA-DR surface expression in a dose-dependent manner, with up to a 4-fold increase. Stable, but elevated, levels of expression were observed for monocytes transfected with HiPe. In summary, our results indicate that transfection with LipoRM is more favorable than Dhar4 and HiPe due to its low immunogenicity, even at higher concentrations.

### 3.4. Whole-Blood Transfection with siRNA and miRNA Downregulates HLA-DR

**Figure 5:**
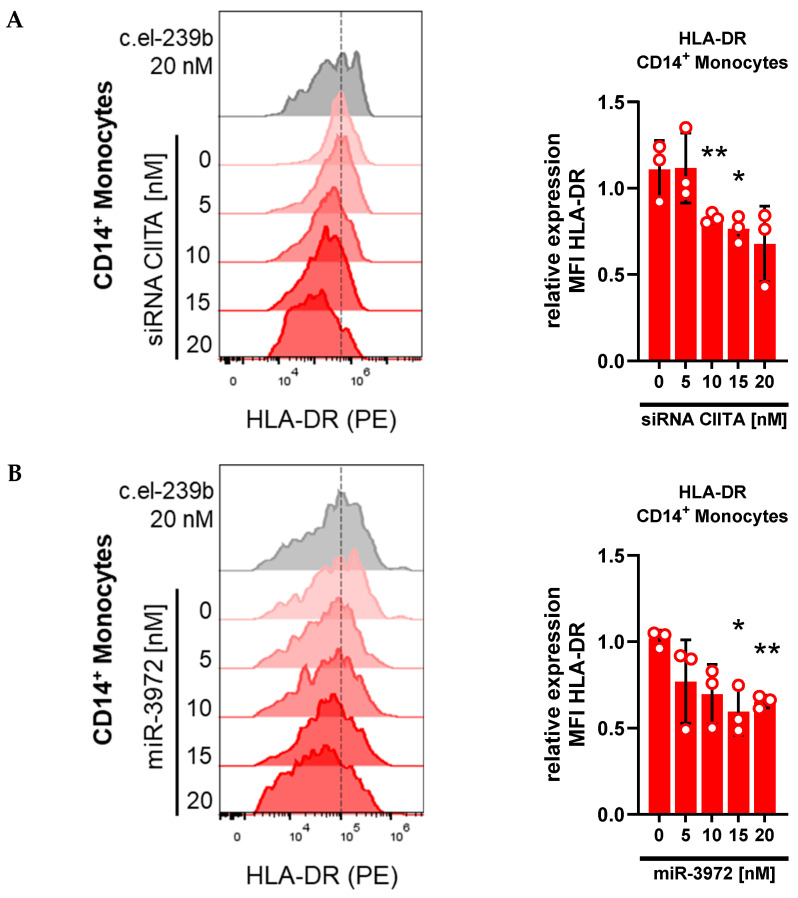
Dose-dependent HLA-DR downregulation in monocytes upon treatment with siRNA and miRNA. (**A**) Median fluorescence intensity (MFI) of HLA-DR expression 72 h after transfection with increasing concentration of siRNA *CIITA* and LipoRM. Bar plot normalized to mock transfected cells (*c.el-239b*). (**B**) Median fluorescence intensity (MFI) of HLA-DR expression 72 h after transfection with increasing concentration of miR-3972 and LipoRM. Bar plot normalized to mock transfected cells (*c.el-239b*). Values for no miRNA (0 nM) refer to untransfected cells. One sample *t*-test * *p* < 0.05; ** *p* < 0.01 compared to mock transfected cells. Values are presented as mean ± standard deviation (SD); n = 3.

One function of monocytes is linking the innate with the adaptive immune system by presenting processed antigens to T-lymphocytes. Therefore, monocytes acquire antigens through phagocytosis and endocytosis. The resulting peptides are processed intracellularly via the major histocompatibility complex II (MHC-II) pathway and displayed in the extracellular space on HLA-DR [25]. To investigate the effectiveness of our *ex vivo* transfection model, we performed a dose-dependent knockdown experiment using specific siRNA against *CIITA,* the main transcription regulator of the MHC-II pathway [25]. Whole blood was transfected with increasing doses of siRNA *CIITA* in combination with LipoRM, followed by a monocytic HLA-DR expression measurement using spectral flow cytometry (Figure 5A). Flow cytometric data showed that cell surface HLA-DR levels in monocytes decreased in a dose-dependent manner when *CIITA* was knocked down. In a further step, whole blood was transfected with an miRNA predicted to target HLA-DR. Applying the miRNA-target gene prediction databases TargetScan [26] and miRmap [27], mir-3972 was identified as miRNA with the highest score to target *HLA-DRA* (TargetScan context score = −0.74 [26]; miRmap score = 97.47 [27]), encoding for the HLA-DR α-chain, a crucial subunit of the HLA-DR complex [28]. Using increasing concentrations of mir-3972 mimic for transfection, we observed a dose-dependent decrease in HLA-DR expression on the monocytes, indicating an miRNA-dependent degradation of *HLA-DRA* mRNA. Taken together, these results confirm the establishment of a functional whole-blood ex vivo model using lipid-based transfection reagents for targeted knockdown in primary monocytes.

## 4. Discussion

Different challenges exist when attempting to chemically transfect isolated primary immune cells. One must address poor transfection efficiency, low survival rates, and often unintended immune stimulation of transfected cells [24,29,30,31]. Although transfection in isolated primary immune cells has the advantage of achieving a better understanding of certain intracellular mechanisms, the physiological relevance of these findings is often limited because immune cells interact with each other through different soluble mediators (e.g., cytokines) [32] and cell–cell interactions (e.g., MHC—T-Cell receptor) [33]. Establishing *ex vivo* transfection protocols offers a suitable alternative to isolated *in vitro* experiments to overcome some of these challenges. Thus, we investigated three different cationic lipid formulations designed for RNAi in whole blood from healthy donors.

To assess the toxicity of the selected reagents, cell viability 72 h post-transfection across leukocyte subpopulations was determined. The miRNA *c.el-239b* without a target in the human transcriptome was selected as a mock sequence to minimize cargo-associated toxicity and to assess the potentially harmful effects of the lipid formulations. Interestingly, none of the selected carriers induced significant toxicity in any of the subpopulations. Even at the concentration of 20 nM *c.el-239b*, the percentage of viable cells remained high (>80%). This low toxicity can be explained by the innate nature of lipid formulations. Liposomal-based carriers consist of hydrophilic and hydrophobic components that allow fusion with lipid bilayers with minimal disruption of the membranes [34]. Furthermore, it is important to consider that the *ex vivo* condition provides an environment close to physiology, which we hypothesize to be beneficial for cell survival. Moreover, no additional isolation techniques other than the use of hirudin-coated tubes for the collection of blood samples have been applied. According to the study of Bexborn et. al, hirudin offers an advantage over other anticoagulants like heparin or EDTA due to its lower toxicity [35].

Transfection efficiency is a critical parameter for the success of any transfection experiment. Primary cells are generally considered to be hard-to-transfect cells due to their sensitivity and lower proliferative capacity compared to immortalized cell lines [36,37]. Therefore, different approaches and modifications to transfection methods have been developed to enhance efficiency in nucleic acid delivery. Engineering nanoparticles or using nucleofection are some examples that have proven to be potential solutions [36,38]. In this study, we aimed to develop a chemical transfection method *ex vivo*. Whole blood was treated with any of the three selected reagents and a fluorescent-labeled miRNA, which served as a traceable marker at a single-cell resolution. Our results revealed that phagocytes, CD15^+^ granulocytes, and CD14^+^ monocytes took up and expressed the labeled miRNA. This observation is consistent with reports proposing that endocytosis and phagocytosis participate in the uptake of the positively charged lipid–nucleic acid complexes [3,39]. Interestingly, the transfection efficiency of CD14^+^ monocytes exceeded 80% despite isolated primary cells generally being considered difficult to transfect [40,41]. This raises the question of whether isolation procedures influence the sensitivity of these cells to transfection. Primary monocyte isolation is typically carried out by density gradient centrifugation followed by anti-CD14 magnetic bead selection. This procedure consists of multiple isolation and washing steps that lead to different molecular characteristics and immunophenotypic behavior and, therefore, potentially influences the ability to absorb lipoplexes [42,43]. On the other hand, incubation in whole blood may enhance the efficiency of chemical transfection methods. The physiological environment may benefit leukocytes and positively affect their ability to take up loaded lipid complexes. To assess potential donor-dependent variation, we calculated the average standard deviation by first determining the standard deviation for each concentration and then computing the mean of these values across all tested concentrations. We observed a trend to larger variations in transfection efficiency for Dhar4 (average standard deviation 13.1% ± 3.7%), followed by HiPe (average standard deviation 7.1% ± 4.8%) and LipoRM (average standard deviation 4.4% ± 1.9%). Although statistical analysis revealed no significant differences between the three reagents, it should be noted that the sample size in our study is small, and as such, not all potential outcomes are accounted for. Therefore, donor-dependent variation always remains a possibility. The second substantially transfected leukocyte population was CD15^+^ granulocytes. LipoRM and Dhar4 demonstrated transfection efficiencies of up to 66% and 59%, respectively. HiPe, on the other hand, was ineffective for delivery into granulocytes. Considering the short lifespan and high susceptibility to activation of isolated granulocytes [44], the observed transfection efficiency *ex vivo* presents a promising approach for RNAi experiments, e.g., in neutrophils. Typically, transfection experiments in neutrophils are conducted using nucleofection, which provides acceptable transfection efficiency. However, performing transient knockdown experiments is limited due to poor survival [45]. Keeping granulocytes under *ex vivo* conditions may be beneficial regarding their survival and outcome of RNAi-based transfection studies. The question arises as to why we observed slightly reduced efficiency with Dhar4 in monocytes and significantly reduced efficiency with HiPe in granulocytes. Potential answers to this question could provide the composition of the three reagents, but due to the proprietary nature of their formulation, a proper interpretation is challenging. Furthermore, up to 20% of CD19^+^ B-lymphocytes and CD56^+^ NK cells demonstrated expression of Alexa Fluor 555 after transfection with Dhar4 and LipoRM, respectively. Lymphocytes are generally regarded as challenging to transfect and show lower transfection efficiencies when using lipid-based reagents. For instance, *in vitro* transfection of NK92 cells, an immortalized NK cell line, using LipoRM resulted in low transfection efficiency and minimal gene silencing [46]. While both populations showed uptake of the lipoplexes, it remains to be evaluated whether this uptake has functional significance.

When working with immune cells, activation is one pivotal parameter. In the present study, we evaluated stimulation-specific surface markers, CD80 and HLA-DR, on monocytes [18,19]. The use of LipoRM appeared to be more favorable than the other two reagents. CD80, as well as HLA-DR expression, remained at a low level, close to the untreated cells. HiPe and Dhar4 led to an increase of CD80 and HLA-DR, suggesting that some component of their formulation may have immune-stimulatory effects. Various factors can trigger such an immune response. The recognition of liposomal complexes by immune cells in the extracellular space can lead to increased secretion of interferon-γ, which promotes the innate immune response [47]. Another explanation is the endosomal detection of nucleic acids by intracellular toll-like receptors (TLRs) leading to subsequent heightened activity [48]. Moreover, other receptors, like C-type lectin receptors and complement receptors, have been reported to be involved in lipid-mediated immune activation, resulting in a pro-inflammatory state [49]. Moreover, monocytes in whole blood treated with HiPe and Dhar4 exhibited large variations of CD80 and HLA-DR expression after transfection. This may be attributed to donor-dependent variations in the expression of the above-mentioned receptors. Furthermore, a study conducted by Forsbach et al. assessed various siRNA delivery systems for their potential to trigger pro-inflammatory cytokine release in isolated peripheral blood mononuclear cells (PBMC). Dharmafect 1 and HiPe samples exhibited increased secretion of type I interferons and TNF-α, both of which are recognized as monocyte activators [50]. The extent of released pro-inflammatory cytokines, along with counteracting anti-inflammatory mechanisms, may explain the high variability in the Dhar4 and HiPe samples.

Taken together, our results show that LipoRM turned out to be superior to HiPe and Dhar4 in terms of transfection efficiency and immune stimulation. However, the present study has some limitations and does not encompass the full range of possibilities for ex vivo transfection. Other methods, such as electroporation or the application of modified small nucleic acids, offer alternatives with potentially better outcomes regarding the assessed endpoints. Furthermore, underlying donor- or patient-dependent factors or predispositions may impact the transfection or future therapy outcomes. Nevertheless, this study could serve as a template for evaluating new RNAi-based treatments and provide an option for preliminary testing in a relevant, close-to-physiology setting before progressing to time-intensive and costly *in vivo* models.

## 5. Conclusions

Our study assessed three commercially available lipid-based transfection reagents for the delivery of miRNA/siRNA into primary leukocytes within whole-blood cultures. CD14^+^ monocytes were identified as a predominantly transfected population among leukocytes. Evaluation of toxicity, transfection efficiency, and immune activation revealed Lipofectamine RNAiMAX as the most suitable reagent for the lipid-based transfection of whole-blood monocytes, due to its low cytotoxicity, high transfection efficiency, and minimal induction of monocyte activation. The functionality of the model was confirmed by demonstrating targeted knockdown of HLA-DR using both siRNA and miRNA. This study provides an effective transfection model for the investigation of cellular mechanisms in monocytes in a physiologically relevant context.

## Data Availability

The original contributions presented in this study are included in the article. Further inquiries can be directed to the corresponding author(s).
